# Experimental and Numerical Investigation of Compressive Membrane Action in GFRP-Reinforced Concrete Slabs

**DOI:** 10.3390/polym15051230

**Published:** 2023-02-28

**Authors:** Gobithas Tharmarajah, Su Taylor, Desmond Robinson

**Affiliations:** 1 Department of Civil Engineering, Faculty of Engineering, Sri Lanka Institute of Information Technology, New Kandy Road, Malabe 10115, Sri Lanka; 2 School of Natural and Built Environment, Queen’s University Belfast, Belfast BT9 5AG, UK

**Keywords:** compressive membrane action, GFRP-reinforced slabs, corrosion, durability, bridge decks

## Abstract

Experimental and numerical analyses of eight in-plane restrained slabs (1425 mm (length) × 475 mm (width) × 150 mm (thickness)) reinforced with glass fiber-reinforced polymer (GFRP) bars are reported in this paper. The test slabs were installed into a rig, that provided 855 kN/mm in-plane stiffness and rotational stiffness. The effective depths of the reinforcement in the slabs varied from 75 mm to 150 mm, and the amount of reinforcement changed from 0 to 1.2% with 8, 12, and 16 mm bar diameters. A comparison of the service and ultimate limit state behavior of the tested one-way spanning slabs shows that a different design approach is necessary for GFRP-reinforced in-plane restrained slabs that demonstrate compressive membrane action behavior. Design codes based on yield line theory, which considers simply supported and rotationally restrained slabs, are not sufficient to predict the ultimate limit state behavior of restrained GFRP-reinforced slabs. Tests reported a higher failure load for GFRP-reinforced slabs by a factor of 2, which was further validated by numerical models. The experimental investigation was validated by a numerical analysis, and the acceptability of the model was further confirmed by consistent results obtained by analyzing in-plane restrained slab data from the literature.

## 1. Introduction

Bridge deck slabs exposed to extreme environmental conditions deteriorate due to steel corrosion. Extensive corrosion caused by de-icing salts and corrosive environments have raised concerns about the service life of steel-reinforced deck slabs and other steel-reinforced structures [1]. Considering their exposure to corrosive conditions, bridge decks are often constructed using several steel protection methods such as high-quality concrete, epoxy coated steel, and waterproofed bridge decks in an attempt to enhance their durability [2,3]. However, the failures of these methods have raised concerns about their long-term reliability [4].

Replacing steel with corrosion-resistant reinforcement such as stainless steel or fiber-reinforced polymer (FRP) bars in concrete structures can be beneficial. Strong fibers, durability, and lightweight are the attractive features of FRP bars. The initial cost, brittleness, low stiffness of some fibers, and inflexibility once produced were found to be the major disadvantages [5]. Although FRP materials have been used extensively to strengthen existing structures, their application as an internal reinforcing material in concrete have not gained much appreciation due to the perceived drawbacks such as larger crack widths, higher deflection resulting from lower modulus of elasticity, and catastrophic failure due to FRP rupture [6]. Nevertheless, continuous research on the application of FRP as a primary reinforcement has helped to develop current design guidelines and codes such as ACI 440.1R-15 [7], the IStructE interim guidelines [8], and the Canadian Highway Bridge Design Code [9].

The application of FRP reinforcement in concrete structures has been studied extensively. In the early 1990s, studies [10,11,12] focused on evaluating the behavior of FRP-reinforced beams and slabs to examine their conformity to the then-existing design codes. Further experimental investigation by Michaluk et al. [13], Hassan et al. [14], El-Salakawy & Benmokrane [15], Zhang et al. [16], and Benmokrane et al. [17] on GFRP, CFRP, steel, and hybrid-reinforced simply supported slabs and beams showed that FRP-reinforced elements demonstrate higher deflection and larger crack widths than steel-reinforced members. This was attributed to the lower modulus of elasticity of FRP bars, particularly the commonly used GFRP. Increasing the amount of reinforcement to enhance the axial rigidity and reduce the deflection in FRP-reinforced concrete structures was the general recommendation in the design codes. Although this is appropriate for simply supported slabs, the approach can be conservative for in-plane restrained slabs. Most bridge deck slabs are in-plane restrained (e.g., Y-beam and W-beam bridge decks). Therefore, it is vital that the benefits of compressive membrane action be considered in bridge deck design.

When a slab is restrained for in-plane expansion, the compressive membrane action phenomenon influences the serviceability and ultimate limit state behavior of the concrete slab. Compressive membrane action (CMA) in short-span-reinforced concrete slabs has been extensively discussed in the literature [18,19,20,21,22,23,24,25,26,27,28,29,30,31,32,33]. Research based on laboratory experiments and field applications have demonstrated [20,31,32,34] that these slabs require a considerably lower amount of steel than simply supported slabs. However, investigation on the influence of compressive membrane action in FRP-reinforced concrete beams and slabs is limited due to lesser number of research. The benefits of CMA in steel-reinforced restrained slabs have been investigated extensively and these can be found elsewhere [18,19,20,21,22,23,24,25,26,27,28,29,30,31,32,33,35,36]. CMA can benefit the design in several ways.

- It can reduce the amount of reinforcement used in deck slabs [19,20,30,31,37]. Northern Ireland bridge design specifications [38] and Highways England design code CD 360 [39] recommend much lower reinforcement percentages (0.3%) than the traditionally required amount of steel for slabs that benefit from CMA.
- The designer can focus on the serviceability limit state requirements as the ultimate limit state is governed by CMA-induced concrete crushing failure. Therefore, for GFRP-reinforced slabs, explicit design for failure criteria is not necessary.

Although the benefits of CMA have been discussed for over 100 years, designers still have a limited understanding of empirical design methods as they are insufficiently explored in major design codes [9,38,39,40]. Engineers have a choice to adopt when designing in-plane restrained slabs that benefits from CMA.

The study presented in this paper experimentally and numerically investigate the behavior of in-plane restrained GFRP-reinforced slabs and provides a basis for consideration of CMA in design approach of FRP-reinforced flexural elements.

The test specimens were compared for their serviceability and ultimate limit state behavior changing various reinforcement parameters. The test slabs used one type of concrete mix design to keep the concrete strength parameter unchanged. Position of the reinforcement, namely either conventional two layers or single mid depth reinforcement; amount of reinforcement, spacing between reinforcement bars are the main reinforcement parameters considered.

The tests programme observed serviceability and ultimate limit state behavior of GFRP-reinforced in-plane restrained slabs. Experimentally tested slabs were modelled using commercially available nonlinear finite element analysis tool to further evaluate the behavior of in-plane restrained slabs. It can be seen from the test results that the nonlinear finite element analysis tool can predict the ultimate failure load of the test slabs and a further evaluation of the model using the data obtained from the literature validate the usability of the model for in-plane restrained slabs.

## 2. Experimental Investigation

Eight in-plane restrained full scale slabs (1425 mm (length) × 475 mm (width) × 150 mm (thickness)) reinforced with glass-fiber-reinforced-polymer (GFRP) bars were constructed using high-strength concrete and tested for service and ultimate load state behavior. The test slabs were constructed using a concrete mix with a target cube compressive strength of 65 N/mm^2^. The concrete mix was consistent for all test slabs and typical of that used in bridge decks. The mix design used in this study was based on the concrete mix used in previous research [41]. Concrete cubes of 100 mm × 100 mm were tested in accordance with BS EN 12390-Part 3 [42] to estimate the compressive strength of the concrete and the cylinder strength f_ck_ was estimated for nonlinear finite element analysis using BS EN 1992 [43], which considers cylinder strength to be equivalent to 80% of the cube strength f_ck,cube_ (f_ck_ = 0.8 × f_ck,cube_).

Commercially available GFRP bars were used for the tests. The bars were tested for tensile strength and modulus of elasticity using the test methods suggested by ASTM D3916 [44] with the gripping improvements recommended by Castro and Carino [45]. The measured tensile strength and modulus of elasticity were 682 N/mm^2^ and 67,400 N/mm^2^, respectively, for the GFRP bars.

**Figure 1:**
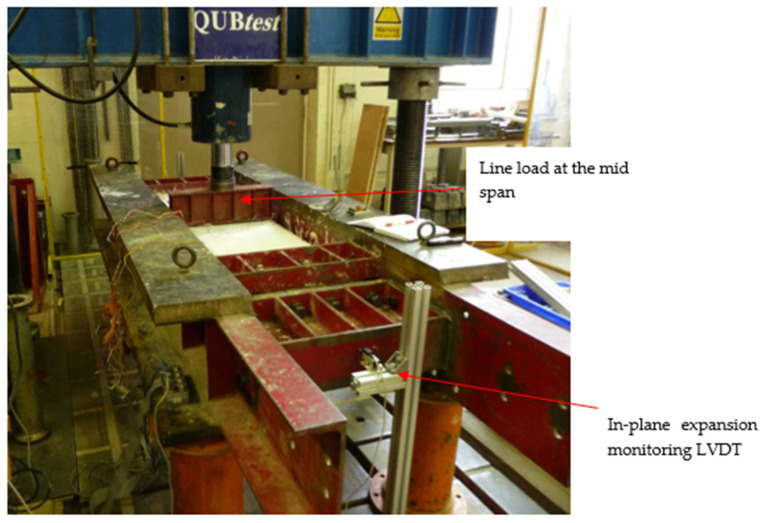
Test load arrangement of the slabs.

**Figure 2:**
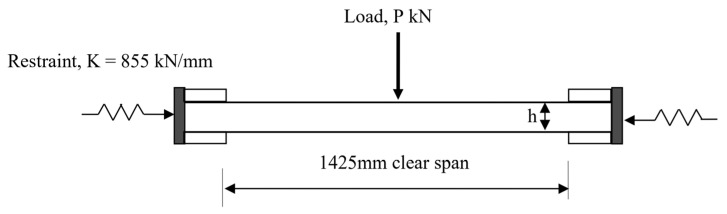
Illustration of the test setup.

The test variables were reinforcement percentage, spacing between bars, effective depth, and bar diameter, as detailed in Table 1. A steel frame with a tested linear stiffness of 855 kN/mm was used to provide an in-plane restraint (Figure 1 and Figure 2). The stiffness was obtained by measuring the axial deformation of the frame for applied axial loads. A similar frame has previously been used by Ruddle [36] and Taylor [41] to investigate arching action in steel-reinforced concrete structures. It was estimated from the relationship developed by Rankin and Long [32] that the frame would provide 70% of a rigid restraint based on the ratio of the in-plane restraint stiffness to the arching stiffness of the slab. Flexural test on GFRP-reinforced slabs for service and ultimate limit state were carried out by fixing the slabs into the steel frame.

Reinforcement amounts of 0, 0.15, 0.60, and 1.2% were selected to investigate the influence of reinforcement percentage in GFRP-reinforced restrained slabs. A lower level of 0.15% was chosen according to the lower limit allowed for a slab by BS EN 1992 [43]. It should be noted that the Canadian code [40] recommends a minimum of 0.25% FRP reinforcement for slabs. However, 0.15% is lower than the minimum recommended by the Canadian code and similar to the minimum recommended level of steel according to BS EN 1992. The upper limit of 1.2% was chosen as it was double the 0.6% reinforcement, which was selected based on steel-reinforced in-plane restrained slabs tested by Taylor et al. [20]. A slab with zero reinforcement was also tested to understand and demonstrate the influence of arching action on the enhancement of the strength and stiffness. The balanced amount of reinforcement required for a flexural member was calculated using the Equation (1) (Equation (7.2.1b) from ACI 440 [7]).

(1) $$\rho_{fb}=0.85\beta_{1}\frac{f_{c}^{\prime }}{f_{fu}}\frac{E_{f\;}\varepsilon_{cu}}{E_{f}\varepsilon_{cu}+f_{fu}}$$

where *f′_c_*—compressive strength of the concrete, *E_f_*—modulus of elasticity of the GFRP, *ε_cu_*—ultimate strain in the concrete, *f_fu_*—design tensile strength of the FRP, and *β*_1_—0.65.

Taking the lowest compressive strength obtained in the tests, and using *β*_1_—0.70 (estimated from Table 6.2 of ACI 440.1R [7]), *f′_c_*—0.8 × 60.4 = 48.32 N/mm^2^, *E_f_*—67,400 N/mm^2^, *ε_cu_*—0.003 and *f_fu_*—0.7 × 682 = 477.4 N/mm^2^, the balanced reinforcement was estimated to be 1.792%. It shall be noted that all test slabs were reinforced with less than the balanced amount of reinforcement. If not the influence of CMA, all the slabs should fail by GFRP rupture. Design guidelines [7] recommend balanced or more than balanced amount of reinforcement to achieve concrete crushing failure as FRP rupture in sections reinforced with less than balanced reinforcement considered catastrophic.

### Instrumentation and Testing

**Figure 3:**
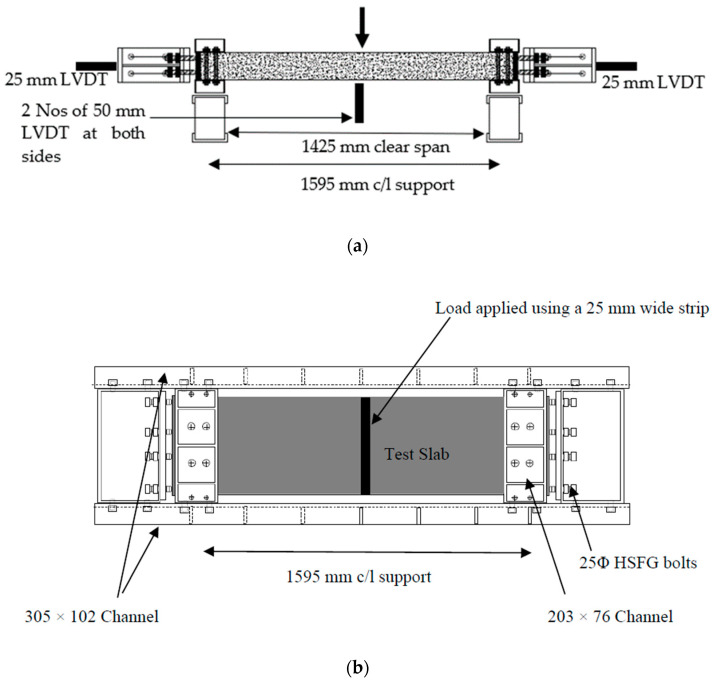
Test set up and test rig. (**a**) Side elevation of the test setup. (**b**) Plan view of the arrangement of the slab inside the test rig.

Test slabs were instrumented to obtain the mid-span vertical deflection of the slabs, in-plane expansion of the steel frame, crack width on test slabs, and strain on GFRP bars. The arrangement of the linear variable differential transformers (LVDT) used to measure deflection is shown in Figure 3. Two 50 mm LVDTs were used to measure the vertical displacement of the test slab directly below the loading point. Two 25 mm LVDTs were placed at the mid-depth of each end-face of the test frame to measure the in-plane expansion. Vibrating wire gauges were attached perpendicular to cracks at the soffit of the test slabs using polyester resin to measure the crack widths and electrical resistance gauges (ERS), gauges were embedded on the reinforcements at the mid span and restrained edge to measure the strain on reinforcement.

Load was applied through a 25 mm × 475 mm plate attached to a stiff beam and bedded on a soft board that prevented any stress concentration. The applied load represented part of an axle load and gives a more onerous representation of the wheel load on a one-way spanning slab as the slab strip would have only a portion of the whole wheel load acting on it. A similar loading method employed to study the flexural behavior of the slabs under flexural conditions can be found in the literature [20,46].

Two service loads equivalent to one-third of the predicted ultimate flexural load were applied initially. The service loads were held for 5 min and unloaded to allow for maximum recovery. Then, the slabs were loaded incrementally by 5 kN steps until failure and data were recorded for each 5 kN increment. The deflection of the slab, in-plane expansion of the frame and strain on the GFRP bars were recorded at each increment of the load.

## 3. Results

GFRP bars demonstrate a low modulus of elasticity and rupture failure. Therefore, the serviceability behavior of the slabs such as deflection, crack width expansion, and reinforcement stress at service load level and failure mode are discussed in this paper with great interest. The peak load at failure and failure mode are also compared.

### 3.1. Deflection

**Figure 4:**
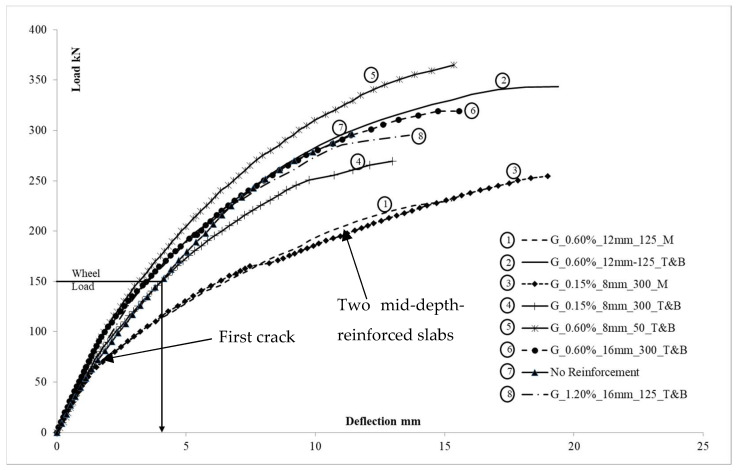
Load vs. deflection behavior of the tested slabs. It can be seen that the ultimate failure load is about two times the maximum tandem system load model 1 (TSLM1) service wheel load of 150 kN.

Load versus mid-span deflection is shown in Figure 4. A linear response was noticed until the formation of the first crack, followed by a non-linear post-cracking behavior. Once cracked, single mid-depth-reinforced slabs showed higher deflection at corresponding loads than conventional double-reinforced slabs. The higher deflection of single mid-depth-reinforced slabs was caused by the loss of concrete stiffness resulting from crack formation and the position of the reinforcement at the mid-depth.

Once a crack is formed in the tension zone, the behavior of the concrete slabs was influenced either by the position and the stiffness of the reinforcement or the stiffness of the membrane arch due to the in-plane restraints. All the double-reinforced slabs, independent of the amount of reinforcement, showed similar load versus deflection behavior up to 200 kN. The deflection values of the test slabs are compared in Table 2 for the EC1 [47] maximum tandem system load model 1 (TSLM1) service wheel load of 150 kN and also at failure load. This is an onerous comparison as the slab strip would have only a portion of the whole wheel load acting on it. The two slabs with single mid-depth reinforcement showed higher deflection exceeding the span/250 limit. The allowable deflection for flexural members is limited to span/250 for steel-reinforced concrete slabs and span/500 if the slab deformation would cause any damages to the structure underneath. Therefore, span/250 is considered for bridge decks. Similar limits can be found in ACI-318-19 [48] for steel-reinforced slabs. Considering an allowable deflection of 5.7 mm for a span of 1425 mm, it can be seen from Figure 4 that two-layer GFRP-reinforced slabs satisfy the deflection criteria for a service load of 150 kN.

The higher stiffness and lower deflection of the unreinforced slab compared to the single mid-depth-reinforced slabs can be attributed in part to the higher concrete compressive strength compared to other slabs. All the test slabs were tested using the same frame with identical in-plane stiffness. Therefore, lower deflection in two-layer-reinforced slabs can be attributed to the reinforcement position, which provided better control of the crack-width expansion as noticed in the restrained slabs. The tests demonstrate that the conventional two layers of reinforcement is necessary to satisfy the deflection limit criteria. Independent of the amount of reinforcement, all the test slabs with two layers of reinforcement satisfy the deflection limits.

### 3.2. Crack Pattern and Crack Width

**Figure 5:**
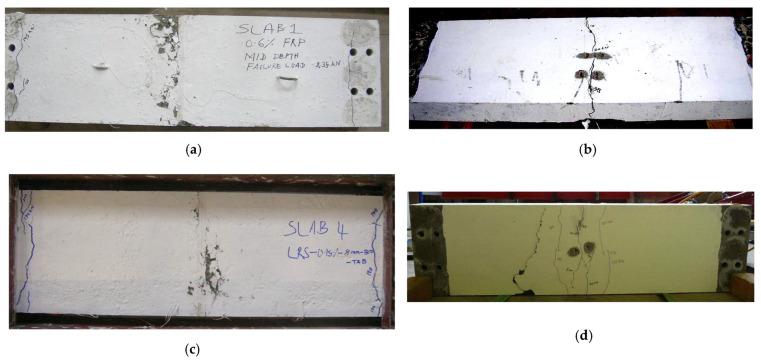
Crack patterns in slabs. (**a**) Typical crack pattern on the top surface of the unreinforced and 0.6% single mid-depth-reinforced slabs. (**b**) Typical crack pattern on the soffit of the unreinforced and 0.6% single mid-depth-reinforced slabs. (**c**) Typical crack pattern on the top surface of the two-layer-reinforced test slabs and 0.15% single mid-depth-reinforced slabs. (**d**) Typical crack pattern on the soffit of the two-layer-reinforced test slabs and 0.15% single mid-depth-reinforced slabs.

**Figure 6:**
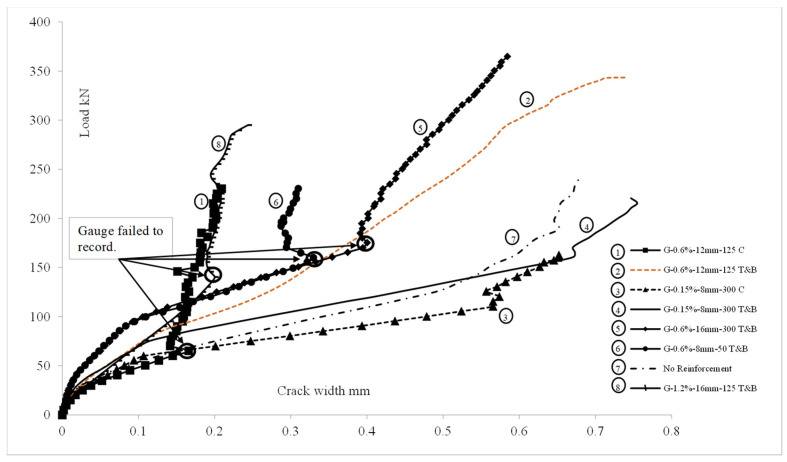
Crack width expansion with load.

Two types of crack pattern were observed during the tests on GFRP-reinforced slabs. Two-layer-reinforced concrete slabs showed one type of crack pattern and 0.6% single mid-depth-reinforced and unreinforced slabs showed another type of crack pattern. Only three cracks were noticed in unreinforced and single mid-depth-reinforced slabs (Figure 5a,b). The first crack appeared directly below the loading point, and the crack width increased with load increment as shown in Figure 6. Two more cracks were noticed at the top surface closer to the fixed ends at higher loads.

In addition to the primary crack on the soffit, a few additional cracks were noticed at the soffit parallel to the first crack at higher load levels in two-layer-reinforced (Figure 5c,d) slabs. The expansion of the crack width of the primary crack on the test slabs is plotted against the applied load in Figure 6. The crack width on the test slabs at various load levels is given in Table 2. The measured crack width at the maximum equivalent tandem system load model 1 service load of 150 kN is compared against the allowable value for all test slabs.

Design codes such as Eurocode restrict the crack widths to less than 0.3 mm for steel-reinforced concrete structures. However, considering the corrosion-resistant nature of GFRP bars, the Canadian code [9] recommends up to 0.5 mm crack width for GFRP-reinforced structures exposed to extreme environmental conditions, and the Japanese guidelines [49] limit the crack width to 0.5 mm based on aesthetic concerns.

Our observations of the crack patterns and crack width expansion suggest that the conventional two-layer reinforcement method can control the rapid development of the depth and width (Figure 6) of the cracks. Unreinforced slabs and the slabs with single mid-depth reinforcement exhibited almost double the crack width of other slabs at a load of 65 kN.

Crack width exceeded 0.5 mm for the 0.15% GFRP-reinforced slab with the conventional two layers at 150 kN. The results show that two layers of 0.6% GFRP reinforcement is required to meet the recommended service behavior in concrete slabs. Maximum crack widths for 0.6% two-layer-reinforced slabs were below 0.33 mm at 150 kN, which was just above the allowable crack width for steel-reinforced structures and well within the allowable 0.5 mm crack width for FRP-reinforced structures.

### 3.3. Stress Recorded on GFRP Bars Using ERS Gaugge

Unlike simply supported slabs, the load on an in-plane restrained slab is carried by both flexural and arching phenomena. As a result, the load is partially carried by the flexural strength of the reinforced concrete. Stress was calculated using the strain data and a modulus of elasticity of 67,500 N/mm^2^ for GFRP bars (Table 2). The design tensile stress of GFRP bars was estimated to be 477.4 N/mm^2^ considering the ultimate tensile stress obtained from GFRP bar tests multiplied by an environmental reduction factor of 0.7. Table 6.2 of ACI 440.1R [7] recommends an environmental design factor of 0.7 for GFRP-reinforced structures exposed to earth and weather. It can be seen from Table 2 that the maximum wheel load stress on GFRP bars was less than the design tensile stress of 477.4 N/mm^2^.

**Figure 7:**
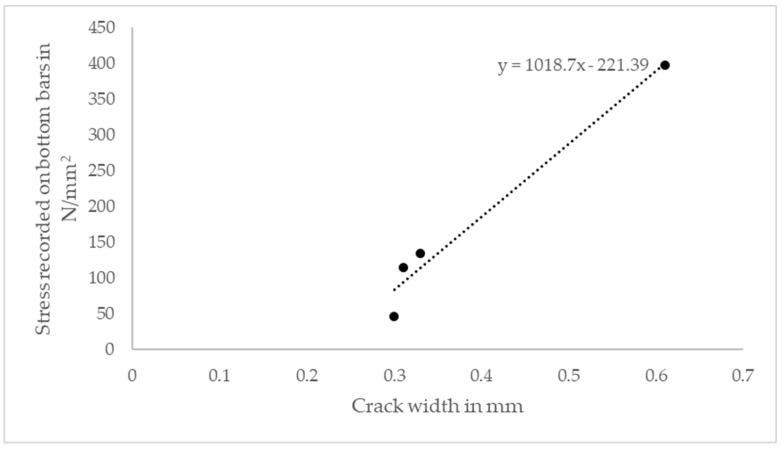
Crack width vs. stress on bottom GFRP bars at 150 kN load.

Due to the creep rupture failure of FRP materials, the service load level stress on GFRP bars is limited to 20% of the design tensile stress, as given in Table 7.4.1 of ACI 440.1R [7]. If the effect of creep due to sustained and cyclic loads is considered, the stress on GFRP bars must be maintained below 95.48 N/mm^2^. Among the three slabs reinforced with the conventional two layers of 0.6% GFRP reinforcement, test slabs with 50 mm spacing (G-0.6%-8 mm-50_T&B) demonstrate stress below 95.48 N/mm^2^. The stress on GFRP bars is influenced by the crack width, as is discussed in the literature [50]. A linear response can be observed when plotting the crack width against the reinforcement stress, as shown in Figure 7.

The higher reinforcement stress in the 0.15% GFRP-reinforced slab and 0.6% single mid-depth-reinforced slab (G-0.6%-12 mm-125-M) can be attributed to the lower amount of reinforcement in 0.15% GFRP-reinforced slabs and the reduction in the availability of depth for flexural capacity in slab G-0.6%-12 mm-125-M due to mid-depth reinforcement.

### 3.4. Failure Load and Failure Criteria

Table 3 summarizes the failure load and the types of failure of the test slabs. All slabs were reinforced with less than a balanced amount of reinforcement except the unreinforced slab. All the test slabs failed by concrete crushing independent of the amount of reinforcement and carried a higher load than the TSLM1 wheel load of 150 kN. Concrete crushing failure is preferred as the failure mode rather than GFRP rupture under flexural loading conditions. Unlike reinforced concrete slabs, the unreinforced concrete slab collapsed into two pieces at the concrete crushing failure. Concrete crushing failure in unreinforced slab and slabs reinforced with a less-than-balanced amount of reinforcement indicate the significant influence of CMA in determining the failure mode of restrained slabs. It was also interesting to observe that the unreinforced slab continued to carry a load even after the formation of a crack at the soffit due to the compressive membrane effect.

The test slabs demonstrated good strength that was in excess of the maximum EC1 [47] service wheel load of 150 kN. The failure load is compared with the load predicted using Equation (7.2.2f) of ACI 440.1R [7] in Table 3. The difference between the predicted failure load and the actual failure load is quite significant. The theoretical equation predicts zero load carrying capacity for the unreinforced slab. However, the unreinforced slab carried a load of up to 296.7 kN. The difference indicates the necessity of including CMA in the load calculation of GFRP-reinforced slabs as ultimate failure load and failure mode in deck slabs such as M- and W-beam bridge decks are governed by compressive membrane action. Additionally, the experimental investigation indicates that it requires about 1/3 of the balanced amount of reinforcement to achieve the preferred service and ultimate limit state behavior in deck slabs similar to the one tested.

## 4. Nonlinear Finite Element Analysis

A commercially available DIsplacement ANAlyser (DIANA) [51] finite element code was used for the non-linear finite analysis of the tested slabs. After an initial comparing of the responses of the plane stress and shell element models, plane stress analysis was carried out using 8-noded quadrilateral element CQ16M.

### 4.1. Modelling Approach

#### 4.1.1. Material Models

The total strain fixed crack model was adopted for numerical analysis. Linear tensile stiffening was assumed for the post-peak tensile behavior. The Thorenfeldt [52] compression model was used for the non-linear hardening behavior of concrete. Although higher stiffness for load versus deflection behavior was observed for all the test models analyzed, the FE model analyzed using the total strain fixed crack model with Thorenfeldt compression behavior predicted the ultimate failure load with good accuracy.

FRP reinforcement was modelled with elastic and Von Mises plastic material models. A fully bonded criterion was chosen for the bond slip behavior in the slabs. The linear elastic property of the steel reinforcement was its modulus of elasticity of 67,400 N/mm^2^, and the non-linear plastic property was 682 N/mm^2^ of yield/rupture stress. An ideal plastic condition was considered, although FRP is brittle in nature. This was due to the limited availability of brittle models in the analysis code. However, the slab tests showed that the reinforcement stress was within the maximum rupture stress due to the influence of CMA. Therefore, the plastic characteristic of the reinforcement material considered has a negligible influence on the behavior.

#### 4.1.2. Numerical Discretization

Saatci and Vecchio [53] recommended an aspect ratio of less than 2 to get better accuracy. However, the selection was not validated quantitatively. A more comprehensive study by Duchaine and Champliaud [54] showed that the error could increase with the aspect ratio. The error was calculated from a comparison between the analytical and nonlinear analysis of bending stresses at three selected locations for a quadrilateral 2D element. The error was 12% for the aspect ratio of 20:1. However, the error was less than 1% for the 6:1 aspect ratio. If 47.5 mm × 5.4 mm was considered as the element size, the error could be around 2% due to the aspect ratio. Therefore, in an attempt to reduce the error to less than 1%, an aspect ratio of 4:1 was chosen for NLFEA analysis. The element size was fixed to 40 mm × 10 mm. The details of the model used for the analysis is given in Table 4.

#### 4.1.3. Solution Method

In this analysis, a regular Newton—Raphson method was used for the iteration. The stiffness matrix of the system is calculated at the end of each iteration step. This could reduce the number of iterations required to reach convergence. However, as this method updates the stiffness matrix for each step, this would require more time to analyze.

#### 4.1.4. Convergence Criteria

Among the available four convergence norms, including force norm, displacement norm, energy norm, and residual norm, this analysis used energy norm to determine the convergence. A convergence tolerance ratio of 0.001 was selected based on a few preliminary analyses performed with nonlinear models. It has been found that the selected tolerance ratio provides acceptable accuracy, and thus it was used for the nonlinear investigation carried out on the test slabs investigated in this paper.

### 4.2. Nonlinear Finite Element Analysis Results

In the tested slab system, the slabs were clamped into a steel frame (Figure 1, Figure 2 and Figure 3). The frame simulates the actual condition of the bridge deck slab in beam-and-slab bridges where the edge beams provide restraint to the lateral expansion of the deck slab. In order to model similar conditions, the lateral stiffness of the frame was used. The stiffness of the test frame was found to be in the range of 855 kN/mm from the axial test carried out on the frame. Therefore, in this method, the restrained edge was assigned with a lateral stiffness equivalent to the steel frame. The elastic springs used to provide the lateral stiffness were only deployed in the compression regime as the top parts of the restrained edge experienced tension. The restraint was applied to the part of the slab edge that experienced only compressive stress. This enabled an equal amount of stiffness to be assigned to each node along the compression zone depth. The symmetric nature of the support conditions, and the loading at the mid-span were considered while developing the geometry of the model and half of the slab was modelled and analyzed for the plane stress analysis. Table 5 shows a comparison between the experimental failure load and the prediction via a nonlinear finite element analysis (NLFEA). The ratio between the experimental failure load (P_T_) and estimated failure load (P_FEM_) using NLFEA shows good agreement between the two.

Christiansen [54] compared four simply supported and four in-plane restrained slab strips to demonstrate the compressive membrane action in those slabs. The in-plane restraint was provided using a steel frame that represented more closely a fixed end condition. The behavior of all the slab strips was studied under a concentrated load applied at the mid-span. The investigation parameters were span/depth ratio of the test slabs, concrete strength, and stiffness of the in-plane restraint. Roberts [55] carried out tests using a wide range of parameters such as concrete strength, span to depth ratio, and reinforcement percentage. The slabs were provided with a lateral restraint using a concrete surround within which the slabs were placed on knife edges. This represented a simply supported slab with in-plane expansion restraint. The tests were carried out using a four-point test system where the bending moment would be constant at the middle quarter of the span. Although Roberts tested 36 specimens, this analysis covered only a few selected slabs due to the similar nature of many of the slabs, where all the slabs had almost the same span to depth ratio and the main investigation parameters were concrete strength and reinforcement ratio.

Lahlouh and Waldron [26] investigated three one-way slab strips where the amount of lateral restraint was changed by using different-sized supporting walls. Three slab and wall models were tested while keeping the reinforcement percentage and concrete strength constant. The slabs were supported by walls of 100, 200, and 300 mm thickness, respectively. The reinforcement ratio was unchanged for the slab (0.54%), and the reinforcement ratios in the walls were 1.34, 0.38, and 0.24% for 100, 200, and 300 mm wide walls, respectively. The side walls were of the same height, and the slabs had the same clear span. Experimental results published by Taylor et al. [19] were also used to validate the proposed nonlinear model. The original experiments were carried out to investigate the strength and behavior of steel-reinforced in-plane restrained slabs with various concrete strengths, reinforcement percentages, in-plane stiffnesses, and positions of reinforcement. The concrete strength varied between 30 N/mm^2^ and 100 N/mm^2^. Two different stiffness values were used, and two different reinforcement positions were investigated.

Taylor and Mullin [45] tested six slabs to investigate the influence of CMA on steel-reinforced and GFRP-reinforced slabs. Among the six slabs, three were reinforced with steel and another three were reinforced with GFRP. In these two groups, two slabs were laterally restrained, and one was simply supported. All the slabs were reinforced with a 0.5% single reinforcement layer at the mid depth. The concrete strength and type of reinforcement were the parameters investigated. The one-way spanning models were loaded at the mid span until failure. The test setup and type of modelling were similar to the tests by Taylor et al. [19]. The authors of this paper also adopted a similar test arrangement with different reinforcement parameters.

It can be seen from Table 6 that the ratio between the test results (P_T_) and NLFEA predictions (P_FEA_) show a good correlation. A comparison of the test results with the NLFEA predictions for the slabs tested by the authors is shown in Table 5, and the comparison shown in Table 6 for the data obtained from the literature shows that the proposed NLFEA model was suitable to analyze both simply supported (as demonstrated for slabs tested by Taylor and Mullin [45]) and in-plane restrained slabs under the influence of compressive membrane action.

## 5. Discussion

Compressive membrane action in in-plane restrained slabs has been under investigation for a considerable time. However, in-depth studies on the subject are limited only to steel-reinforced slabs. Understanding compressive membrane action in reinforced concrete slabs can benefit the construction of flexural members by reducing the amount of reinforcement [31], replacing two layers of reinforcement with one mid-depth reinforcement [20], and even in some instances completely removing steel from concrete slabs [55]. The research into compressive membrane action in steel-reinforced slabs has resulted in its inclusion in several design codes [38,39,56].

The experimental investigation carried out on seven in-plane restrained GFRP-reinforced concrete slabs and one unreinforced slab shows that in-plane restrained slabs demonstrate far more strength compared to the required wheel load of 150 kN, including unreinforced slabs, and the failure load was 80–100% higher than the flexural strengths predicted by design guidelines [7]. The increased failure load of restrained slabs and the strength of unreinforced slabs can be attributed to the arching action that is not considered in many design codes when determining the strength of in-plane restrained FRP-reinforced concrete slabs. When the behavior of unreinforced restrained slabs and GFRP-reinforced restrained slabs are compared, it can be seen that the strength can almost exclusively be achieved through arching action. This shows the influence of the arching action in restrained slabs and the necessity of taking arching action into consideration when designing in-plane restrained FRP-reinforced concrete slabs.

A comparison of the deflection, crack width expansion, and stress of GFRP bars indicated that GFRP reinforcement in restrained slabs is required to control crack width, deflection, and reinforcement stress in the service limit state. The conventional two layers of reinforcement show acceptable deflection limits compared to single-mid-depth-reinforced slabs. Although a single mid-depth reinforcement was demonstrated to be sufficient for steel-reinforced in-plane restrained slabs, the same cannot be extended to GFRP-reinforced slabs due to the lower modulus of elasticity of GFRP bars. Test slabs reinforced with two layers of 0.6% GFRP reinforcement or higher satisfy both the service and ultimate limit state requirements. A 0.6% GFRP bar is a direct replacement for steel with GFRP bars being on an equal volume basis as recommended by the NI specifications [38] and CD 360 [39]. This study expands the possibility of using GFRP bars in in-plane restrained slabs as a direct replacement for steel on a volume basis when the slabs satisfy the requirement for CMA.

**Figure 8:**
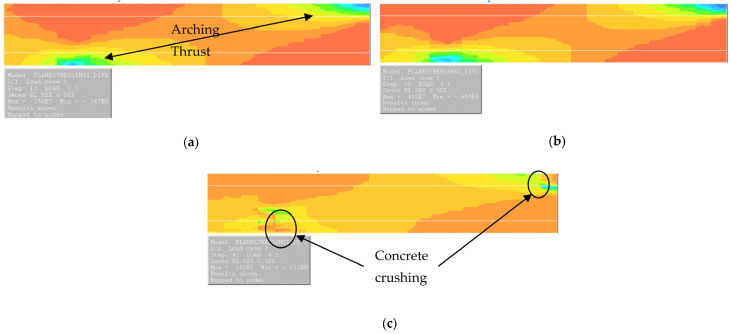
Stresses on a slab. (**a**) Stress distributions along the length of the slab at service load level. This figure shows only half the model of the slab. (**b**) Stress distributions along the length of the slab at the peak load. (**c**) Stress distributions along the length of the slab at the post failure stage.

NLFEA carried out on GFRP-reinforced concrete slabs demonstrates that the proposed model satisfactorily predicts the behavior of in-plane restrained GFRP-reinforced concrete slabs. The comparison shown in Table 4 and Table 5 demonstrates the good level of compatibility of the model when considering a large spectrum of data of in-plane restrained slabs. Figure 8 shows the stresses on a modelled typical test slab. We can see the formation of an arching thrust in (a), cracked zones at the peak load in (b) and the failure state stresses in (c). A good agreement between the experimental investigation, numerical analysis, and theoretical understanding was established through the study.

## 6. Conclusions

The following conclusions can be drawn at the end of the study:

(1) As has been seen in steel-reinforced concrete in-plane restrained slabs, GFRP-reinforced in-plane restrained slabs also demonstrate the influence of compressive membrane action at the service and ultimate limit state. The strength capacities shown by in-plane restrained GFRP-reinforced and unreinforced slabs above the flexural strengths predicted by design codes. All the test slabs show strength far more than the required wheel load of 150 kN including unreinforced slab. The failure load was 80–100% higher than the flexural strengths predicted by Eurocode and ACI 440.1R. The increased failure load of restrained slabs and the strength of unreinforced slab can be attributed to the compressive membrane action which is not considered in many design codes to determine strength of in-plane restrained reinforced concrete slabs.
(2) The unreinforced slab was tested and compared to demonstrate the real strength of in-plane restrained slabs. The comparison between unreinforced restrained slab and GFRP-reinforced restrained slabs shows that the strength can almost exclusively be achieved through arching action. This shows the influence of compressive membrane action in restrained slabs and the necessity to adopt arching action for consideration while designing in-plane restrained GFRP-reinforced concrete slabs.
(3) GFRP-reinforced in-plane restrained slabs that satisfy the conditions for compressive membrane action as described in CD 360 [38], acceptably satisfy the service and ultimate load state requirements when reinforced with two layers of 0.6% GFRP reinforcement or higher.
(4) Although the unreinforced slab, the slabs reinforced with a single mid depth reinforcement and two layer 0.15% GFRP reinforcement show strength excess of a wheel load, they fail to satisfy serviceability requirements at maximum wheel load of 150kN. These deficits to satisfy serviceability criteria shall be attributed to the lower amount of reinforcement and the position of reinforcement.
(5) NLFEA carried out on tested slabs and numerical analysis of data obtained from the literature clearly indicate the ability of nonlinear finite element models to predict the behavior of restrained slabs and the influence of compressive membrane action. Therefore, it is appropriate to adopt necessary modifications in design codes to incorporate the benefits of compressive membrane action that can significantly benefit GFRP/FRP-reinforced in-plane restrained slabs.

## 7. Recommendation for Future Work

The study reported in this paper evaluated the behavior of GFRP-reinforced in-plane restrained slabs. This investigated the influence of the reinforcement percentage, reinforcement spacing and reinforcement position. However, it is important to study the influence of concrete strength on GFRP-reinforced in-plane restrained slabs. It is also important to analyze the behavior of in-plane restrained slabs reinforced with more than balanced amount of reinforcement and under cyclic loading conditions.

## Figures and Tables

**Table 1:** Details of the test slabs and test conditions.

Test Slabs	Effective Depth (Rebar Spacing)	Reinforcement (%)	Compressive Strength, (^†^ f_ck,cube_ N/mm^2^)	Tensile Strength (N/mm^2^)
G-0.6%-12 mm-125_M	75 mm (125 mm)	0.60	64.7	3.70
G-0.6%-12 mm-125_T&B	119 mm (125 mm)	0.60	68.1	3.44
G-0.15%-8 mm-300_M	75 mm (300 mm)	0.15	69.5	2.52
G-0.15%-8 mm-300_T&B	121 mm (300 mm)	0.15	66.7	4.05
G-0.6%-16 mm-300_T&B	117 mm (300 mm)	0.60	65.7	3.68
G-0.6%-8 mm-50_T&B	121 mm (50 mm)	0.60	60.4	3.96
No reinforcement	No reinforcement	0.00	72.6	3.97
G-1.2%-16 mm-125_T&B	117 mm (125 mm)	1.20	66.3	3.87

^†^ Note: cylinder strength f_ck_ = 0.8 × f_ck,cube_, as recommended in EC2 (BS EN 1992-1-1, Table 3.1 [43]).

**Table 2:** Deflection and crack width expansion of the slabs at different load levels.

Slab	Concrete Strength f_ck,cube_ (N/mm^2^)	Deflection @150kN (mm)	Ratio of Span to Deflection at Service Level	Deflection at FAILURE (mm)	Crack Width @ 65kN in (mm)	Crack Width @ 150kN (mm)	Stress on GFRP Bar @ 150 kN (N/mm^2^)
G-0.6%-12 mm-125_M	64.7	6.57 (43%) *	217	15.27	0.17	-	220.2
G-0.6%-12 mm-125_T&B	68.1	3.49 (18%)	408 > 250 ^$^	19.40	0.09	0.33	134.3
G-0.15%-8 mm-300_M	69.5	6.34 (33%)	225	18.98	0.15	-	184.3
G-0.15%-8 mm-300_T&B	66.7	4.06 (31%)	351 > 250	12.97	0.09	0.61	397.5
G-0.6%-16 mm-300_T&B	65.7	3.20 (21%)	445 > 250	15.35	0.05	0.31	114.8
G-0.6%-8 mm-50_T&B	60.4	3.45 (22%)	413 > 250	15.56	0.05	0.30	46.9
No reinforcement	72.6	4.05 (36%)	352 > 250	11.41	0.14	0.57	-
G-1.2%-16 mm-125_T&B	66.3	3.52 (26%)	405 > 250	13.62	0.09	-	262.0

* The percentage to the deflection at failure. ^$^ Allowable span-to-depth ratio.

**Table 3:** Failure load and mode of test slabs.

Slab Model	Reinforcement Percentage (%)	Balanced Reinforcement % Based on ACI 440.1R	Failure Load Predicted by ACI 440.1R (kN)	Failure Load PT kN	Expected Failure Mode	Actual Failure Mode
G-0.6%-12 mm-125_M	0.60	1.85	119.4	235.0	GFRP rupture	Concrete crushing
G-0.6%-12 mm-125_T&B	0.60	1.88	190.1	343.5	GFRP rupture	Concrete crushing
G-0.15%-8 mm-300_M	0.15	1.91	26.7	254.8	GFRP rupture	Concrete crushing/
G-0.15%-8 mm-300_T&B	0.15	1.91	42.8	269.0	GFRP rupture	GFRP rupture Concrete crushing
G-0.6%-16 mm-300_T&B	0.60	1.88	166.2	364.9	GFRP rupture	Concrete crushing
G-0.6%-8 mm-50_T&B	0.60	1.79	171.3	319.2	GFRP rupture	Concrete crushing
No reinforcement	0.00	2.00	0.0	296.7	GFRP rupture	Concrete crushing
G-1.2%-16 mm-125_T&B	1.20	1.90	331.4	295.1	GFRP rupture	Concrete crushing

**Table 4:** Details of the numerical model.

Model Type	Element Type	Mesh Size	Material Models	Solution Method	Loading Type
Plane Stress	Eight-node quadrilateral isoparametric plane stress element CQ16M	40 mm × 10 mm	Total Strain Fixed Crack model using Threnfeldt compression criteria for concrete	Newton—Raphson	Displacement control at the mid span

**Table 5:** Comparison of the experimental failure load with nonlinear finite element analysis.

Slabs	Compressive Strength of Concrete (N/mm^2^)	Experimental Failure Load (P_T_)	Failure Load by NLFEA (P_FEA_)	P_T_/P_FEA_
G-0.6%-12 mm-125_M	64.7	235.0 kN	217.0 kN	1.08
G-0.6%-12 mm-125_T&B	68.1	343. 5 kN	289.3 kN	1.19
G-0.15%-8 mm-300_M	69.5	254.8 kN	213.8 kN	1.19
G-0.15%-8 mm-300_T&B	66.7	269.0 kN	232.6 kN	1.16
G-0.6%-16 mm-300	65.7	364.9 kN	280.3 kN	1.30
G-0.6%-8 mm-50	60.4	319.2 kN	268.0 kN	1.19
No reinforcement	72.6	296.7 kN	219.7 kN	1.35
G-1.2%-16 mm-125_T&B	66.3	295.1 kN	302.2 kN	0.98

**Table 6:** Validation of the NLFEA model using data obtained from the literature.

	Author	L (mm) × h (mm) × d (mm)	f_ck,cube_ (N/mm^2^)	% As	P_b_	P_FEM_	P_T_	P_T_/P_FEM_
1	Christiansen [54]	1829 × 76.2 × 66.7	34.3	0.623	8.43	13.52	11.48	0.85
2		1524 × 76.2 × 66.7	32.3	0.623	10.07	15.48	14.44	0.93
3		1524 × 88.9 × 79.4	28.3	0.623	12.02	19.69	18.02	0.92
4		1524 × 88.9 × 79.4	39.1	0.623	12.22	23.80	19.76	0.83
RB10	Roberts [55]	1463 × 51 × 42.6	50.4	0.556	6.39	19.20	18.72	0.98
RB11			24.7	0.556	6.25	13.20	11.86	0.90
RB12			32.8	0.741	8.33	16.80	16.22	0.97
RB13			30.2	0.741	8.30	16.40	13.14	0.80
RB14			49.7	0.926	8.45	18.80	18.50	0.98
RB15			24.1	0.926	10.10	14.80	13.96	0.94
RB17			53.3	0.578	10.51	18.80	16.88	0.90
RB24			51.8	0.371	4.29	19.20	18.52	0.96
RB25			26.3	0.371	4.24	12.00	14.16	1.18
H-100	Lahlouh and Waldron [26]	2500 × 150 × 121	71.8	0.540	81.20	84.00	84.70	1.01
H-200			78.7	0.540	81.70	108.00	109.70	1.02
H-300			64.4	0.540	80.50	120.00	143.10	1.19
1	Taylor et al. [19]	1425 × 150 × 104	31.2	0.680	91.5	141.7	136	0.96
2			40.8	0.680	93.4	157.8	145	0.92
3			64.5	0.680	94.7	206.5	175	0.85
4			82.2	0.680	94.7	239.9	187	0.78
5			101.1	0.680	94.7	271.3	192	0.71
9			89.3	0.680	94.7	276.7	252	0.91
10		1425 × 150 × 0	90.5	0.000	00.0	210.0	200	0.95
11		1425 × 150 × 75	96.8	0.680	68.3	229.1	223	0.97
14		1425 × 150 × 104	39.5	0.680	93.3	165.2	195	1.18
15		1425 × 150 × 104	60.9	0.680	94.7	213.6	211	0.99
S-40-LR	Taylor and Mullin [45]	1425 × 150 × 75	41.0	0.5	69.2	135.3	130	0.96
S-70-LR			85.0	0.5	72.8	225.8	210	0.93
G-40-LR			38.6	0.5	65.7	131.2	145	1.11
G-70-LR			67.9	0.5	68.6	185.8	200	1.07

## Data Availability

Not applicable.
